# Prognostic and Clinical Value of the Systemic Immune-Inflammation Index in Biliary Tract Cancer: A Meta-Analysis

**DOI:** 10.1155/2022/6988489

**Published:** 2022-11-17

**Authors:** Xiulan Peng, Xia Wang, Li Hua, Rui Yang

**Affiliations:** ^1^Department of Oncology, The Second Affiliated Hospital of Jianghan University, the Fifth Hospital of Wuhan, Wuhan, Hubei 430000, China; ^2^Department of Pharmacy, The Second Affiliated Hospital of Jianghan University, the Fifth Hospital of Wuhan, Wuhan, Hubei 430000, China; ^3^Department of General Medicine, The Second Affiliated Hospital of Jianghan University, the Fifth Hospital of Wuhan, Wuhan, Hubei province, 430050, China; ^4^Department of Vascular Surgery, The Second Affiliated Hospital of Jianghan University, the Fifth Hospital of Wuhan, Wuhan, Hubei 430000, China

## Abstract

Previous studies that explored the prognostic and clinical value of the systemic immune-inflammation index (SII) in biliary tract cancer (BTC) had inconsistent results. We conducted this meta-analysis to evaluate the prognostic and clinicopathological role of the SII in biliary tract cancer. Combined analysis demonstrated that high SII levels had worse overall survival (HR = 1.92, 95% CI: 1.66–2.21, *p* < 0.001) than those with low SII levels. And an elevated SII was associated with lymph node metastasis (OR = 1.44, 95% CI = 1.18‐1.76; *p* < 0.001), TNM stage (OR = 1.49, 95% CI = 1.05‐2.13; *p* = 0.028), and vascular invasion (OR = 1.49, 95% CI = 1.05‐2.13; *p* = 0.028). Conversely, no significant association between a high SII and sex or tumor differentiation was found. Our findings demonstrate that high SII levels were correlated with unfavorable survival outcomes among patients with BTC and that they were also correlated with some higher malignancy features of BTC.

## 1. Introduction

Biliary tract cancer (BTC) is a heterogeneous group of biliary malignant tumors and is the second common cancer of the hepatobiliary system [1]. BTC consists of gallbladder cancer, intrahepatic cholangiocarcinoma, and extrahepatic cholangiocarcinoma [2].BTC is associated with chronic inflammation of the biliary tree and hepatic parenchyma. Chronic hepatitis B or C, primary sclerosing cholangitis, gallstones, and certain liver parasites that might cause chronic inflammation are recognized risk factors for BTC [3]. The outcome of BTC is extremely poor because it has no specific symptoms in its early stages and is usually diagnosed at a relatively advanced stage [4]. For patients with advanced BTC, the median overall survival (OS) is no more than 12 months and 5-year survival rate is only approximately 5-15% [5, 6]. The prognosis of individuals with BTC is poor, partially because there are no efficient prognostic biomarkers currently. Therefore, it is essential to identify novel and efficient prognostic markers for BTC that could be used for precise treatment decision-making and to improve the survival of patients with BTC.

Previous studies have indicated that the inflammatory response plays a crucial role in the tumor microenvironment and is a key mediator of tumor development, progression, and metastasis, including BTC [7, 8]. Therefore, peripheral inflammatory cells and calculated ratios as parameters representing the grade of systemic inflammatory response, such as the neutrophil-lymphocyte ratio (NLR), platelet-lymphocyte ratio (PLR), lymphocyte-to-monocyte ratio (LMR), and C-reactive protein levels, have been reported to be efficient prognostic biomarkers for patients with various cancers [9–11]. The systemic immune-inflammation index (SII) is a novel inflammatory index that has been shown to be a useful prognostic indicator in several cancers, including hepatocellular carcinoma and lung, gastric, and colorectal cancers [12–14].

The SII has been a significant prognostic factor in patients with BTC [15–24]. However, the association between SII and survival in BTC is still controversial. Therefore, we conducted a quantitative meta-analysis to estimate the association between SII and the prognosis and clinicopathological factors of patients with BTC.

## 2. Materials and Methods

### 2.1. Search Strategy

This meta-analysis was performed in accordance with the Preferred Reporting Items for Systematic Reviews and Meta-Analysis (PRISMA) guidelines [25]. The study protocol was registered on the PROSPERO database (CRD42022296509; https://www.crd.york.ac.uk/PROSPERO/). We thoroughly searched PubMed, Web of Science, EMBASE, and the Cochrane Library from their inception until 20 Jun., 2022. The literature search strategy for each database was reported in the Supplementary file. Search results were manually examined to identify potentially relevant studies.

### 2.2. Inclusion and Exclusion Criteria

The inclusion studies must meet the following criteria: (1) histopathologically confirmed diagnosis of BTC, (2) reported hazard ratios (HRs) and corresponding 95% confidence intervals (CIs) of SII, (3) the association between SII and OS and/or RFS, (4) definite cut-off value of SII, and (5) only English papers.

Following studies were excluded: (1) reviews, meta-analyses, conference abstracts, letters, and case reports; (2) studies received any anticancer treatment previously; and (3) did not contain sufficient information.

### 2.3. Data Extraction and Quality Assessment

Two examiners (XW and LH) independently extracted the following information: first author's name, publication year, study period, number of cases, study country, age, sex, time of follow-up, histology, treatment, cut-off value of SII, prognostic endpoints (OS or RFS), HRs with corresponding 95% CIs, and clinicopathological characteristics.

The Newcastle-Ottawa scale (NOS) was employed to assess the quality of the selected studies [26]. NOS scores ranged from 0 to 9; if scores were higher than 6, the study was high quality.

### 2.4. Statistical Analysis

The pooled HRs and 95% CIs were calculated to evaluate the correlation between SII and survival of patients with BTC; the random-effects model was explored to combine the data. The Cochran *Q*-test and *I*^2^ test were used to assess statistical heterogeneity across studies [27, 28], *I*^2^ > 50% and *p* < 0.10 indicating substantial heterogeneity. Subgroup analysis was performed according to the sample size, ethnicity, histology, treatment, cut-off value, and cut-off selection method. Odds ratios (ORs) and 95% CIs were used to determine the correlations between the SII and clinicopathological factors in patients with BTC. Sensitivity analysis was conducted to evaluate the reliability of the results. Begg's and Egger's tests were used to quantify publication bias; *p* < 0.05 was considered as statistical significance. Stata version 15.0 (Stata Corporation, College Station, TX, USA) was used to perform the meta-analysis.

## 3. Results

### 3.1. Search Results

**Figure 1:**
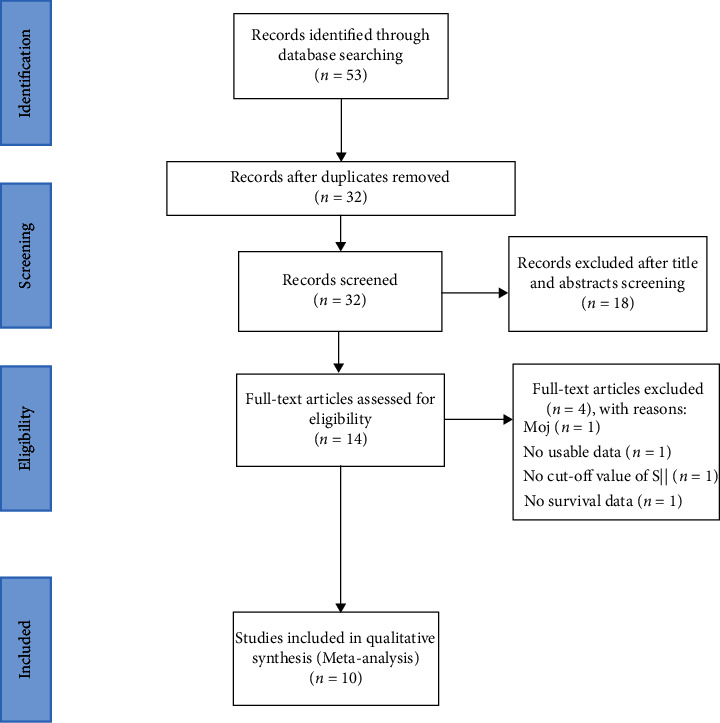
Flow chart of literature search and study selection.

As shown in Figure 1, 53 studies were yielded after the initial search from database, from which 32 papers were retained for full-text review. After screening titles and abstracts, 18 studies were excluded. Finally, 10 studies comprising 2,508 patients were included in the meta-analysis [15–24].

### 3.2. Description of the Included Studies

Table 1 presents the main characteristics of the included studies. All 10 identified studies were retrospective cohorts, and the number of patients in each study ranged from 28 to 691. The included studies were published between 1993 and 2021 and were conducted in seven countries, including one multi-institution (USA, Italy, Australia, China, France, the Netherlands, and Japan) [16], one in Japan [17], and eight in China [14, 18–24]. All studies provided information on the relationship between the SII and OS [15–24], three on RFS [22–24] and seven on clinicopathological features [17–23]. The cut-off values ranged from 331 to 1,450. The NOS scores ranged from 7 to 8(Supplementary file), indicating high-quality studies.

### 3.3. Correlation between SII and OS in BTC

**Figure 2:**
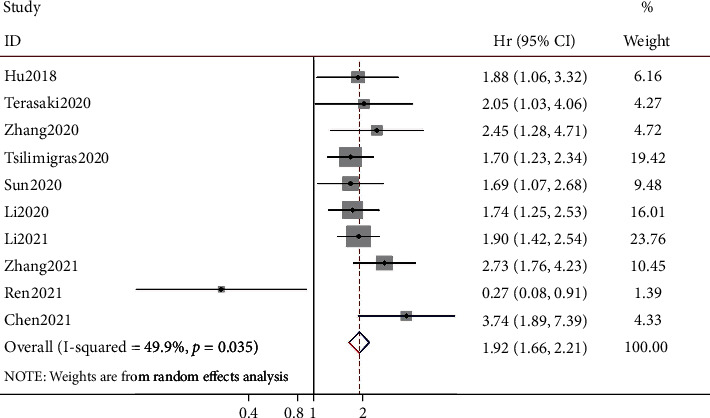
Forest plots of the association between the systemic immune-inflammation index and OS in patients with biliary tract cancer. CI: confidence interval; HR: hazard ratio.

Data showing the relationship between the SII and OS were extracted from all 10 studies including 2,508 patients [15–24]. A random-effects model adopted due to heterogeneity was significant (*I*^2^ = 49.9%, *p* = 0.035; Figure 2 and Table 2). The results of the pooled analysis indicated that high SII was related to poor OS for BTC (HR = 1.92, 95% CI: 1.66–2.21, *p* < 0.001). Subgroup analysis was performed for further investigation based on sample size, ethnicity, histology, treatment, cut-off value, and cut-off selection method. Nonetheless, the results indicated that there was no significant correlation between high SII and sample size or histology, but there was significant heterogeneity in ethnicity (*I*^2^ = 59.5%), cut-off value (*I*^2^ = 68.9%), and cut-off selection method (*I*^2^ = 49.9%) (Table 2).

### 3.4. Correlation between SII and RFS

As there were only three studies that provided RFS data, the pooled results were with low resolution [22–24]. The correlation between SII and RFS in patients with BTC was not analyzed in this study.

### 3.5. Association between SII and Clinicopathological Features

**Figure 3:**
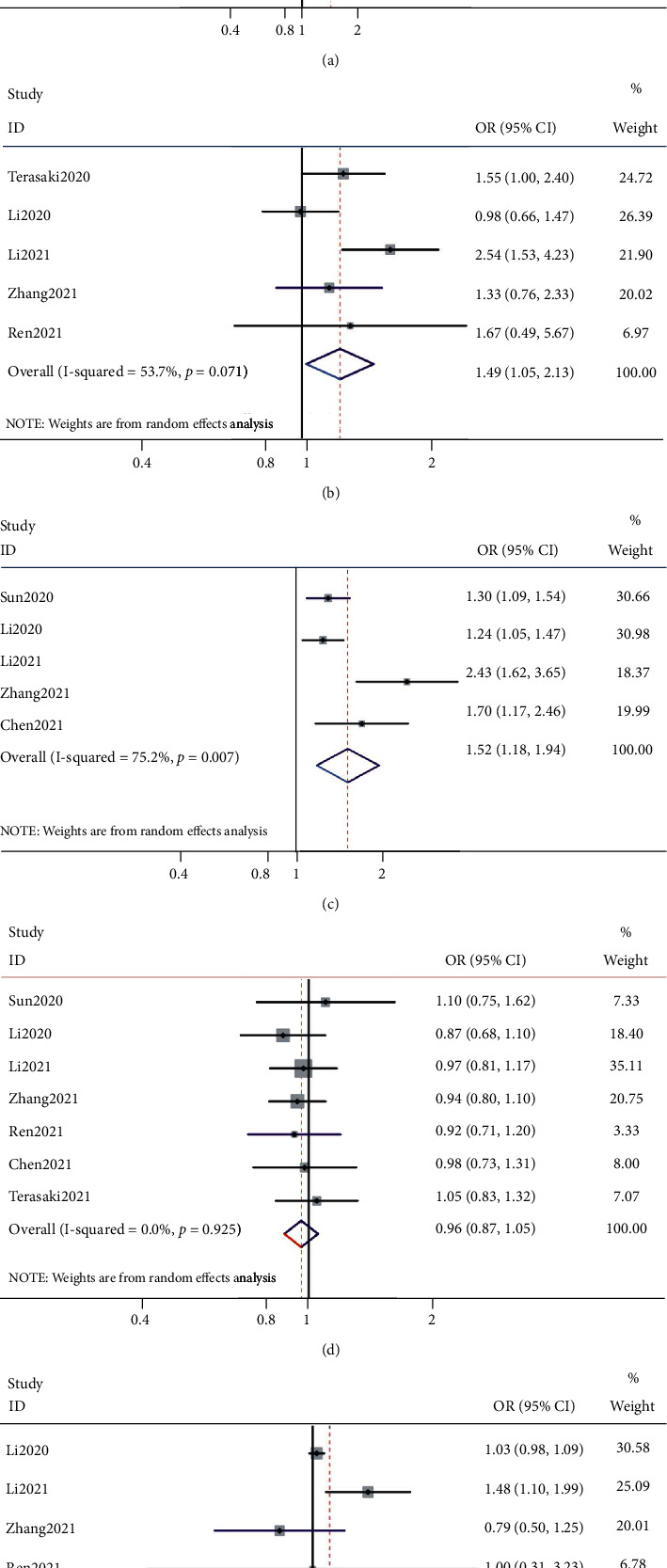
Forest plots of the correlation between the systemic immune-inflammation index and clinical features in biliary tract cancer. (a) Lymph node metastasis (yes vs. no); (b) vascular invasion (yes vs. no); (c) TNM stage (I–II vs. III-IV); (d) sex (male vs. female); (e) tumor differentiation (poor vs. moderate/well).

The association between SII and clinicopathological features was analyzed in seven studies including 1,579 patients [17–23]. The parameters included sex (male vs. female), tumor differentiation (poor vs. moderate/well-differentiated), lymph node metastasis (yes vs. no), TNM stage (I–II vs. III-IV), and vascular invasion (yes vs. no), (Table 3 and Figure 3). The pooled results suggested that a high SII was associated with lymph node metastasis (OR = 1.44, 95% CI = 1.18‐1.76; *p* < 0.001), TNM stage (OR = 1.49, 95% CI = 1.05‐2.13; *p* = 0.028), and vascular invasion (OR = 1.49, 95% CI = 1.05‐2.13; *p* = 0.028). No significant correlation was detected between the SII and sex (OR = 0.96, 95% CI = 0.87‐1.05; *p* = 0.384) or tumor differentiation (OR = 1.06, 95% CI = 0.94‐1.20; *p* = 0.492).

### 3.6. Sensitivity Analysis

**Figure 4:**
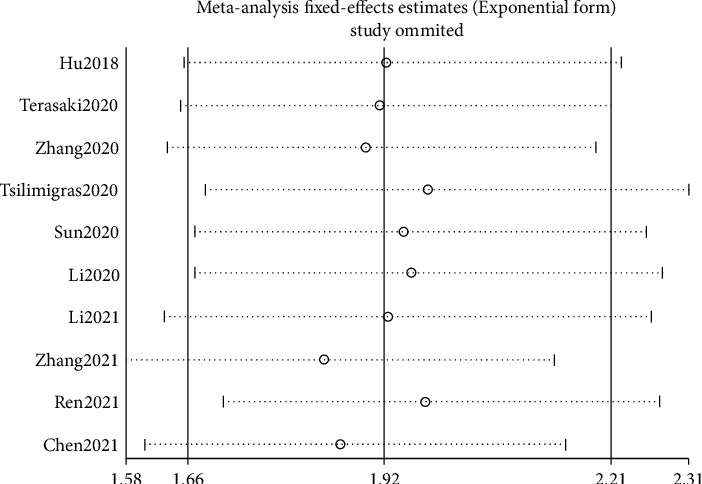
Sensitivity analysis for the OS.

A sensitivity analysis was conducted to evaluate the stability of the pooled HRs for OS, and it showed that the results of this meta-analysis were credible (Figure 4).

### 3.7. Publication Bias

**Figure 5:**
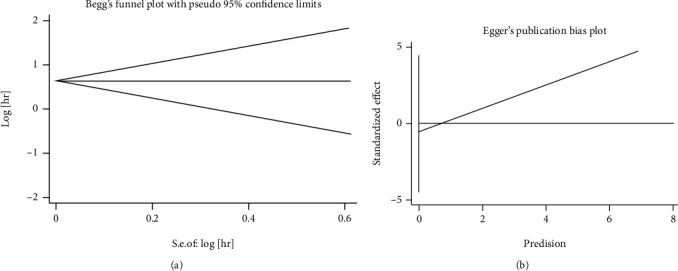
Publication bias. (a) Begg's test for overall survival, *p* = 0.592; (b) Egger's test for overall survival, *p* = 0.710.

Publication bias was assessed by Begg's funnel and Egger's tests, and no significant bias for OS was detected in this meta-analysis (Begg's *p* = 0.592, Egger's *p* = 0.710) (Figure 5).

## 4. Discussion

Due to the pivotal role of chronic inflammatory conditions in the development of BTC, an increasing number of inflammatory markers, such as PLR, LMR, and NLR, have been used in clinical practice as prognostic markers for BTC [29–31]. In this study, survival data from 10 studies which included 2,595 patients were integrated to investigate the clinical value of SII in BTC prognosis. The results demonstrated a relationship between high SII and poor OS in patients with BTC, suggesting that a high SII is a valuable prognostic marker for survival outcomes. Moreover, our studies also observed the association between a high SII and clinical characteristics, including lymph node metastasis, vascular invasion, and advanced TNM stage. Thus, high SII levels were related to tumor progression and invasiveness. The sensitivity and publication bias analysis suggested the results were reliable. Taken together, the current meta-analysis demonstrates the importance of the SII for the prognosis of BTC. So far as we know, this is the first meta-analysis to evaluate the prognostic and clinicopathological significance of the SII in patients with BTC.

Inflammatory cells of the tumor microenvironment are involved in various proinflammatory responses, and the amount of immune cells and other components of the tumor microenvironment is crucial for the initiation, malignant conversion, development, and metastasis of tumors [32]. The SII is determined by neutrophil × platelet/lymphocyte count, and a high SII is caused by changes in these cell counts. Neutrophils can restrict the cytolytic activity of immune cells, secrete all kinds of inflammatory factors, and promote adhesion of circulating tumor cells (CTCs) to target organs, thus resulting in the promotion of tumor progression and metastasis [33–36]. An increase in platelets can stimulate angiogenesis by secreting vascular endothelial growth factor [37]. Platelets can protect tumor cells from immune destruction and promote distant metastasis by inducing CTC epithelial-mesenchymal transition and enhancing their transendothelial migration and metastasis [38, 39]. Lymphocytes play a critical role in cancer immunesurveillance by suppressing tumor growth and progression [40]. Decreased lymphocyte counts can weaken immunological reactions in cancer patients [41]. All these results imply that SII can reflect the balance between host inflammatory and immune status.

The SII reflects the overall status of the patient's immune system and is noninvasive and easily obtained in clinical practice. The prognostic effect of the SII has also been investigated in other types of cancer in a meta-analysis. A recent meta-analysis based on data from 11 studies involving 3,737 patients indicated that a high SII could predict worse survival outcomes and clinical parameters in colorectal cancer [42]. Study from Zhong et al. also showed that a high SII showed prognostic efficiency in multiple solid cancers [43]. A retrospective study including 2,442 patients demonstrated that a high pretreatment SII indicated poor OS and DFS/RFS and was associated with several clinicopathological factors in non-small-cell lung cancer [44]. Wang et al. reported the prognostic value of SII in hepatocellular carcinoma and that priority treatment may be more beneficial for patients with high SII than low SII [45]. In the present meta-analysis, the pooled results indicated that a high SII was significantly related to poor OS, which is consistent with the findings from other cancer studies. In addition, the results demonstrated correlations between a high SII and clinical factors in BTC. Therefore, combined with these findings, the SII may be used for cancer prognosis and help guide clinical decision-making. The measurement of SII is noninvasive, cost-effective, and convenient, implying that SII shows promising clinical efficacy for patients with BTC.

This study has several limitations. First, the cut-off methods and values of SII levels were inconsistent, which may have resulted in heterogeneity. Second, most studies were conducted in Asia, and the results may be more relevant to Asian patients. Third, all the included studies were retrospective, which may have led to heterogeneity among the studies. Further prospective studies on patients are needed in the future.

## 5. Conclusion

This meta-analysis identified 10 studies including 2,508 patients. Survival outcomes demonstrated that a high SII was associated with worse OS in patients with BTC and with clinical features implying higher malignancy of the cancer. These results indicate that SII could play an important role as an effective factor for poor prognosis and guide clinical treatment in patients with BTC. However, as there were several limitations to this study, further high-quality studies are needed to validate our results.

## Figures and Tables

**Table 1:** Baseline characteristics of included studies.

Author Year	Country	Period	Histology	Patients (*n*)	Age (y) median (range)	Sex (M/F)	TNM stage	Cut-off value	Cut-off selection	Treatment	Follow-up (m)	Survival analysis	NOS score
Hu et al. 2019 [15]	China	2012-2017	eCCA	113	68.9 ± 11	75/38	NA	456	ROC	PTBS+^125^I	NA	OS	7
Zhang et al. 2020 [24]	China	2013-2017	iCCA	128	56.19 ± 9.63	70/58	I-III	1027	ROC	Surgery	NA	OS, RFS	7
Tsilimigras et al. 2020 [16]	Multi-institution	2000-2007	iCCA	688	57 (49-60)	416/272	NA	1150	ROC	Surgery	22.3 (10.9-44.6)	OS, CSS	8
Sun et al. 2020 [20]	China	2003-2017	GBC	142	63.06 ± 10.68	60/82	I-IV	600	Harrell miscellaneous	Surgery	NA	OS	8
Li et al. 2020 [22]	China	2009-2017	iCCA	265	57.9	134/131	I-III	450	ROC	Surgery	18 (1-115.4)	OS, RFS	8
Li et al. 2021 [21]	China	2002-2019	GBC	691	55.7 ± 9.5 (32-80)	270/421	NA	510	R package survMisc	Surgery	53.8 (3 m-18 y)	OS	8
Zhang et al. 2021 [23]	China	1993-2015	cHCC-CCA	220	59 (30-81)	162/58	I-IV	331	ROC	Surgery/+TACE	22.1 (1-118)	OS, RFS	8
Ren et al. 2021 [19]	China	2013-2018	iCCA	28	51.5 (46.8-60)	25/3	NA	447.48	ROC	TACE/ablation/chemotherapy/+LT	33.5	OS	7
Chen et al. 2021 [18]	China	2012-2020	GBC	93	62 (32-90)	62/31	I-III	824	ROC	Surgery	14 (2-60)	OS	8
Terasaki et al. 2021 [17]	Japan	2002-2015	eCCA	140	NA	109/31	NA	1450	Log-rank test and K–M curve	Surgery	48.2	OS	7

NA: not available; OS: overall survival; DFS: disease-free survival; NOS: Newcastle-Ottawa scale; ROC: receiver operating characteristics curve; SII: systemic immune-inflammation index; TNM: tumor node metastasis; GBC: gallbladder cancer; iCCA: intrahepatic cholangiocarcinoma; eCCA: extrahepatic cholangiocarcinoma; cHCC-CCA: combined hepatocellular-cholangiocarcinoma; PTBS: percutaneous transhepatic biliary stenting; TACE: transarterial chemoembolization; LT: liver transplantation.

**Table 2:** Subgroup analyses for OS and RFS based on different factors.

Subgroup analysis	No. of studies	No. of patients	Effects model	HR (95% CI)	*p* value	Heterogeneity	
						*I* ^2^ (%)	*p* value
OS							
Histology							
GBC	3	926	Fixed	2.00 (1.58-2.52)	<0.001	48.2	0.145
iCCA	4	1109	Random	1.52 (0.95-2.44)	<0.001	70.4	0.018
eCCA	2	253	Fixed	1.95 (1.26-3.02)	0.03	0.0	0.849
Sample size							
≥140	6	2006	Fixed	1.89 (1.62-2.21)	<0.001	0.0	0.610
<140	5	502	Random	1.49 (0.78-2.84)	0.222	87.1	<0.001
Treatment							
Surgery	7	2087	Fixed	1.90 (1.62-2.22)	<0.001	0.0	0.499
Cut-off value							
≥555	5	1104	Fixed	1.96 (1.57-2.43)	<0.001	21.1	0.280
<555	5	1404	Random	1.74 (1.20-2.53)	0.04	68.9	0.012
Cut-off selection method							
ROC curve	7	1535	Random	1.94 (1.39-2.72)	<0.001	66.0	0.07
Country			—				
China	8	1680	Random	1.96 (1.48-2.58)	<0.001	59.5	0.16
Total	10	2508	Random	1.92 (1.66-2.21)	<0.001	49.9	0.035

CI: confidence interval; HR: hazard ratio; OS: overall survival; DFS: disease-free survival; ROC: receiver operating characteristics curve; SII: systemic immune-inflammation index; PTBS: percutaneous transhepatic biliary stenting; TACE: transcatheter arterial chemoembolization; K–M curve: Kaplan–Meier curve; GBC: gallbladder cancer; iCCA: intrahepatic cholangiocarcinoma; eCCA: extrahepatic cholangiocarcinoma; cHCC-CCA: combined hepatocellular-cholangiocarcinoma.

**Table 3:** Correlations of SII and clinicopathological characteristics.

Clinicopathological features	No. of studies	No. of patients	Effects model	OR (95% CI)	*p* value	Heterogeneity	
						*I* ^2^ (%)	*p* value
Lymph node metastasis (yes versus no)	5	1496	Random	1.44 (1.18-1.76)	<0.001	52.5	0.078
Vascular invasion (yes vs. no)	5	1431	Random	1.49 (1.05-2.13)	0.028	53.7	0.071
TNM stage (I–II vs. III-IV)	4	807	Random	1.52 (1.18-1.94)	0.001	75.2	0.007
Sex (male versus female)	7	1579	Fixed	0.96 (0.87-1.05)	0.384	0.0	0.925
Tumor differentiation (poor versus moderate/well)	5	1384	Fixed	1.06 (0.94-1.20)	0.492	79.1	0.001

CI: confidence interval; OR: odds ratio; SII: systemic immune-inflammation index; BTC: biliary tract cancer.

## Data Availability

The datasets analyzed during this study are available from the corresponding author on reasonable request.

## Supplementary Materials

Supplementary Materials Table S1: the literature search strategy for each database and the detailed items of Newcastle–Ottawa scale in this meta-analysis.
