# Supplementary material for: Prognostic and Clinical Value of the Systemic Immune-Inflammation Index in Biliary Tract Cancer: A Meta-Analysis
Source: J Immunol Res. 2022 Nov 17;2022:6988489. doi: 10.1155/2022/6988489 (PMC9691295; doi:10.1155/2022/6988489)
Supplement: Supplementary Materials — Table S1: the literature search strategy for each database and the detailed items of Newcastle–Ottawa scale in this meta-analysis. [file 6988489.f1.docx]

**Search strategies:**

Pubmed:

#1 "Cholangiocarcinoma"[Mesh] OR "Klatskin Tumor"[Mesh] OR "Biliary Tract Neoplasms"[Mesh] OR "Bile Duct Neoplasms"[Mesh] OR "Gallbladder Neoplasms"[Mesh]

#2 ((((Cholangiocarcinoma[Title/Abstract]) OR (Klatskin Tumor"[Title/Abstract])) OR (Biliary Tract Neoplasms[Title/Abstract])) OR (Bile Duct Neoplasms[Title/Abstract])) OR (Gallbladder Neoplasms[Title/Abstract])

#3 (((systemic immune-inflammatory index) OR (SII)) OR (systemic-immune-inflammation index)) OR (systemic immune-inflammation index)

#4 (#1 OR #2 ) AND (#3)

Web of science:

#1 ((((TS=(Biliary Tract Neoplasms)) OR TS=(Bile Duct Neoplasms)) OR TS=(Cholangiocarcinoma)) OR TS=(Gallbladder Neoplasms )) OR TS=(Klatskin Tumor)

#2 ((((ALL=(systemic immune-inflammatory index)) OR ALL=(SII)) OR ALL=(systemic-immune-inflammation index)) OR ALL=(systemic immune-inflammation index)

#3 (#1) AND (#2)

Cochrane:

#1 MeSH descriptor: [Biliary Tract Neoplasms] explode all trees

#2 (biliary tract cancer):ti,ab,kw OR (Cholangiocarcinoma):ti,ab,kw OR (Klatskin Tumor):ti,ab,kw OR (Bile Duct Neoplasms):ti,ab,kw OR (Gallbladder Neoplasms):ti,ab,kw

#3 (systemic immune-inflammatory index) OR (SII) OR (systemic-immune-inflammation index) OR (systemic immune-inflammation index)

#4 (#1 OR #2) AND (#3)

Embase:

#1 'biliary tract cancer'/exp OR 'bile duct cancer'/exp OR 'bile duct carcinoma'/exp OR 'gallbladder cancer'/exp OR 'klatskin tumor'/exp

#2 'biliary tract cancer':ab,ti OR 'bile duct cancer':ab,ti OR 'bile duct carcinoma':ab,ti OR 'gallbladder cancer':ab,ti OR 'klatskin tumor':ab,ti

#3 'systemic immune inflammatory index'/exp

#4 'systemic immune inflammatory index':ab,ti OR sii:ab,ti OR 'systemic-immune-inflammation index':ab,ti OR 'systemic immune-inflammation index':ab,ti

#5 (#1 OR #2 ) AND (#3 OR #4 )

**Table S1 The detailed items of Newcastle–Ottawa Scale in this meta-analysis**

| Research | Selection | | | | Comparability | | Exposure | | | | NOS Score |
| --- | --- | --- | --- | --- | --- | --- | --- | --- | --- | --- | --- |
|  | Is the case definition adequate? | Representativeness of the cases | Selection of Controls | Definition of Controls | Select the most important factor | study controls for any additional factor | secure record (eg surgical records) | structured interview where blind to case/control status | Same method of ascertainment for cases and controls | Non-Response rate |  |
| Hu^2019^ | 1 | 1 | - | 1 | 1 | 1 | 1 | - | 1 | - | 7 |
| Zhang^2020^ | 1 | 1 | - | 1 | 1 | 1 | 1 | - | 1 | - | 7 |
| Tsilinigras^2020^ | 1 | 1 | - | 1 | 1 | 1 | 1 | 1 | 1 | - | 8 |
| Sun^2020^ | 1 | 1 | - | 1 | 1 | 1 | 1 | 1 | 1 | - | 8 |
| Li^2020^ | 1 | 1 | - | 1 | 1 | 1 | 1 | - | 1 | 1 | 8 |
| Li^2021^ | 1 | 1 | - | 1 | 1 | 1 | 1 | - | 1 | - | 8 |
| Zhang^2021^ | 1 | 1 | - | 1 | 1 | 1 | 1 | 1 | 1 | - | 8 |
| Ren^2021^ | 1 | 1 | - | 1 | 1 | 1 | 1 | - | 1 | - | 7 |
| Chen^2021^ | 1 | 1 | - | 1 | 1 | 1 | 1 | 1 | 1 | - | 8 |
| Terssaki^2021^ | 1 | 1 | - | 1 | 1 | 1 | 1 | - | 1 | - | 7 |
